# Supplementary figures and images for: Pre-exposure to mRNA-LNP inhibits adaptive immune responses and alters innate immune fitness in an inheritable fashion
Source: PLoS Pathog. 2022 Sep 2;18(9):e1010830. doi: 10.1371/journal.ppat.1010830 (PMC9477420; doi:10.1371/journal.ppat.1010830)

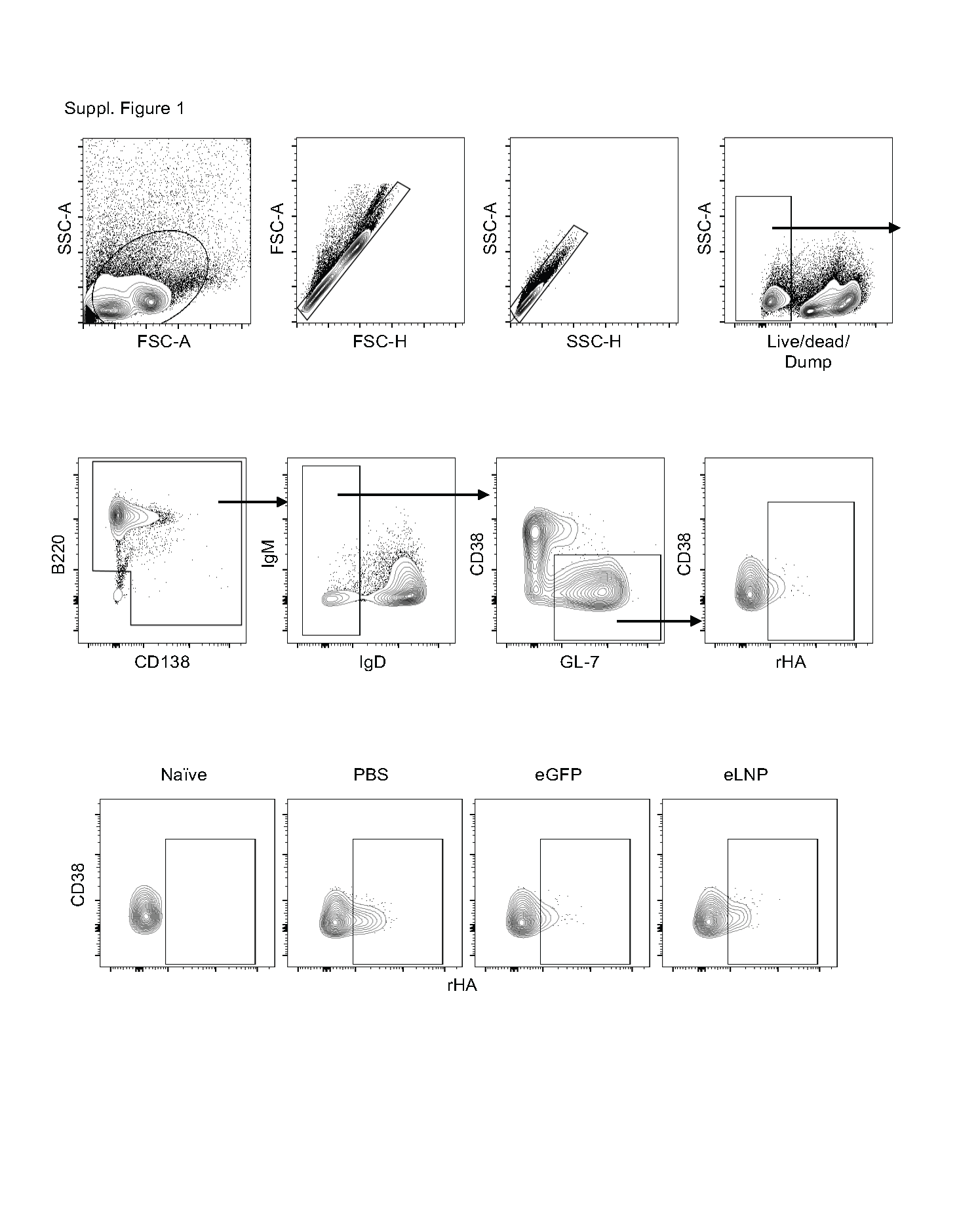

Supplement: S1 Fig — (TIFF) [file ppat.1010830.s001.tiff]

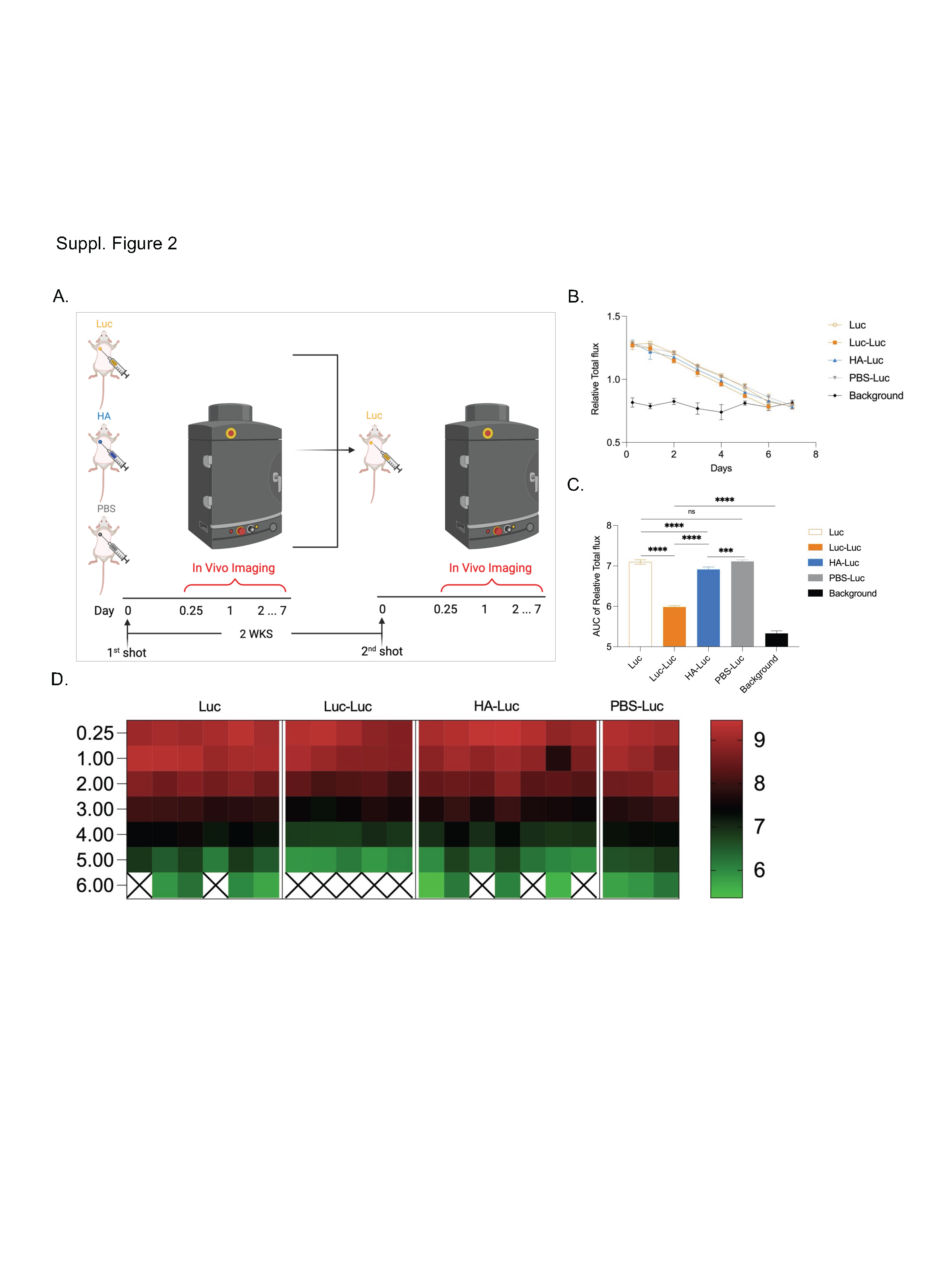

Supplement: S2 Fig — A). Experimental model. Balb/c mice were pre-exposed to PBS, Luc mRNA-LNPs or PR8 HA mRNA-LNPs and imaged using IVIS 6 hours (0.25 day) post inoculation and then every day for 7 days. Two weeks later all the animals were injected in the same spot with Luc mRNA-LNPs and the luciferase signal monitored similarly to the first exposure. B). Relative total flux with time. C). Data from B presented as AUC. D). Total flux values (background subtracted) of each mouse at different time points are shown as log10. X marks mice where the signal was below detection. Data from two separate experiments pooled. One way ANOVA was used to establish significance. ns = not significant. ***p<0.0005, ****p<0.0001. (TIFF) [file ppat.1010830.s002.tiff]

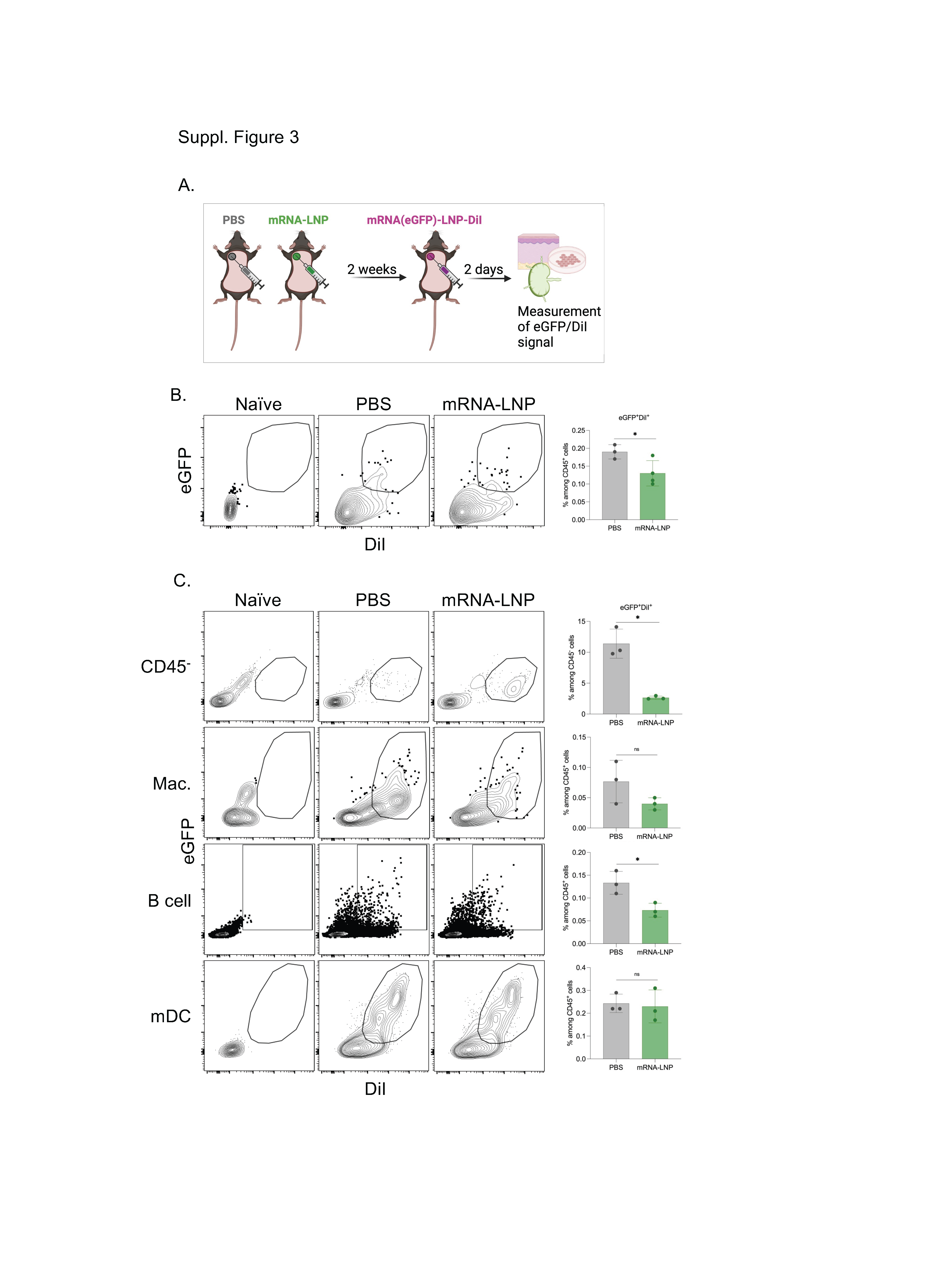

Supplement: S3 Fig — A). Experimental model. Animals were shaved and intradermally inoculated in the left upper spot with either PBS or mRNA-LNP coding for HA. Two weeks later the same areas were injected with mRNA-LNP-DiI coding for eGFP. The injected skin (2cm2) and skin draining lymph nodes were harvested 2 days later and the eGFP and DiI signals determined using flow cytometer. B). Representative flow plots and summary graph on eGFP+DiI+ population of skin DCs (MHCII+ CD11c+) after gating on live cell/Ly-6G-/CD64-. Naïve mice were not injected with mRNA-LNP-DiI coding for eGFP. C). Representative flow plots and summary graphs on eGFP+DiI+ population of SDLNs CD45- cells, macrophages (Mac., CD64+), B cells (MHCII+CD11c-) and mDCs (MHCIIhigh CD11cmid). Each dot represents a separate mouse. The data are from one experiment and are shown as mean ±SD. Welch’s t test was used to establish significance. ns = not significant. *p<0.05. (TIFF) [file ppat.1010830.s003.tiff]

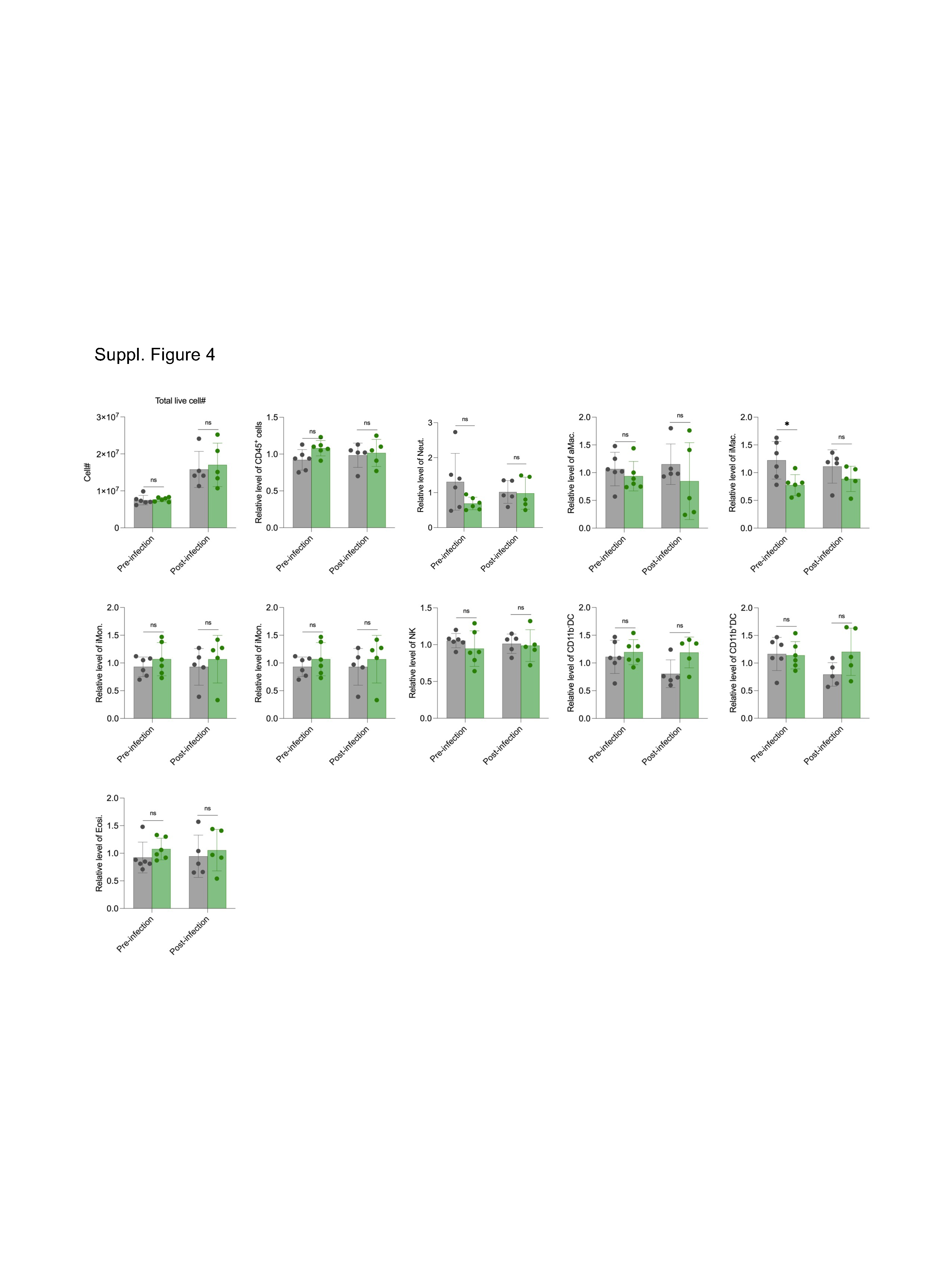

Supplement: S4 Fig — Summary graphs of levels of lung immune cells from pre- and post-influenza infected mice which were pre-exposed to PBS or mRNA-LNP for 2 weeks. Each dot represents a separate mouse. The data were pooled from two experiments. Welch’s t test was used to establish significance. ns = not significant. *p<0.05. (TIFF) [file ppat.1010830.s004.tiff]

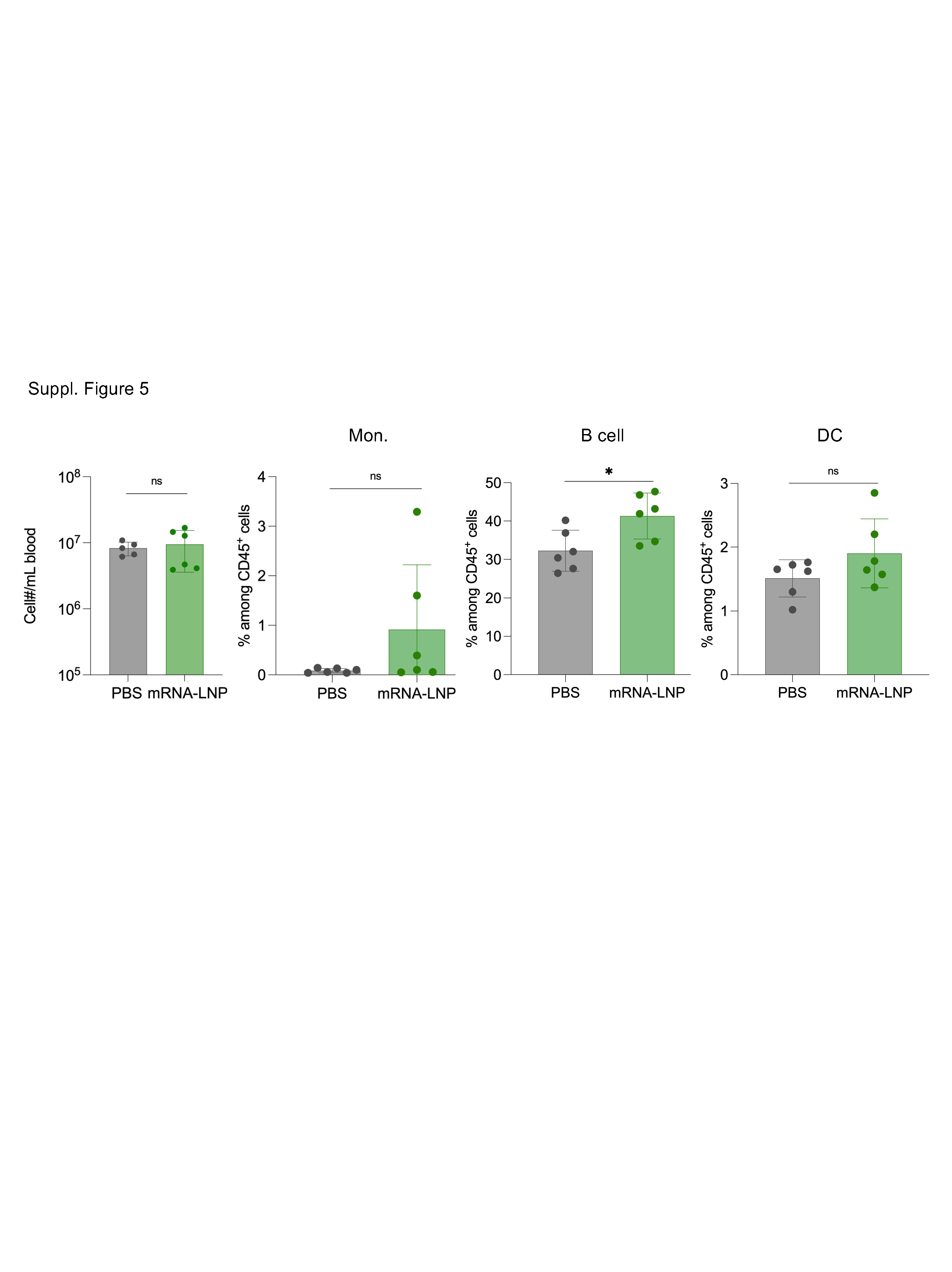

Supplement: S5 Fig — Summary graph of total cell number and major categories of cells among CD45+ cells in PBMCs from mice pre-exposed to either PBS or mRNA-LNP for 2 weeks. Each dot represents a separate mouse. The data were pooled from two experiments. Welch’s t test was used to establish significance. ns = not significant. *p<0.05. (TIFF) [file ppat.1010830.s005.tiff]

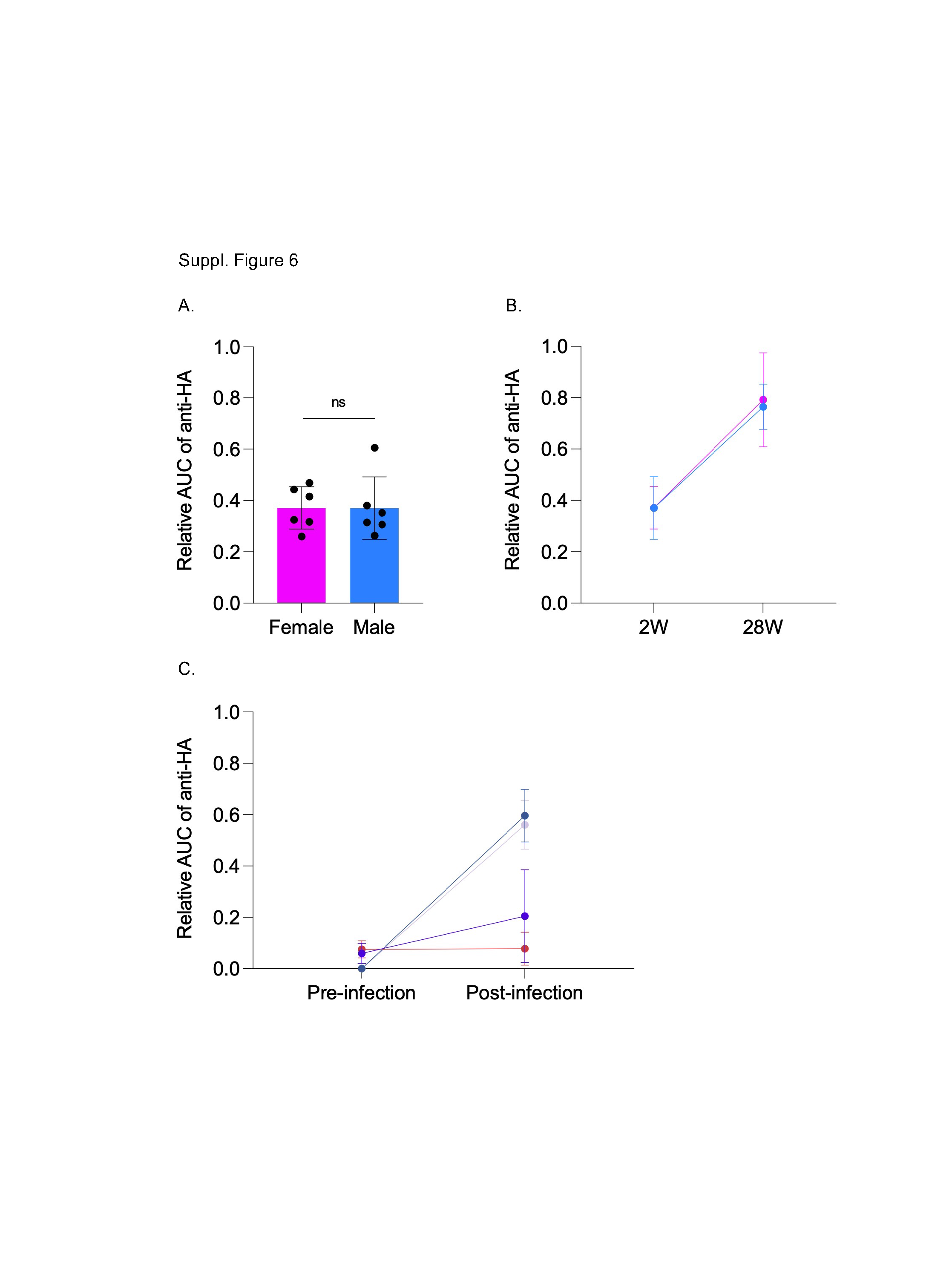

Supplement: S6 Fig — A). Anti-HA antibody levels in parents 2 weeks post inoculation. B). Anti-HA antibody levels in parents 2 weeks and 28 weeks post inoculation. C). Anti-HA antibody levels in the 1st litters prior- and 4 weeks post-infection. (TIFF) [file ppat.1010830.s006.tiff]
